# Supplementary material for: Venetoclax Overcomes Sorafenib Resistance in Acute Myeloid Leukemia by Targeting BCL2
Source: Biology (Basel). 2023 Oct 16;12(10):1337. doi: 10.3390/biology12101337 (PMC10603903; doi:10.3390/biology12101337)
Supplement: Supplementary file 1 [file biology-12-01337-s001.zip › Supplementary Materials.pdf]

Supplementary Figures

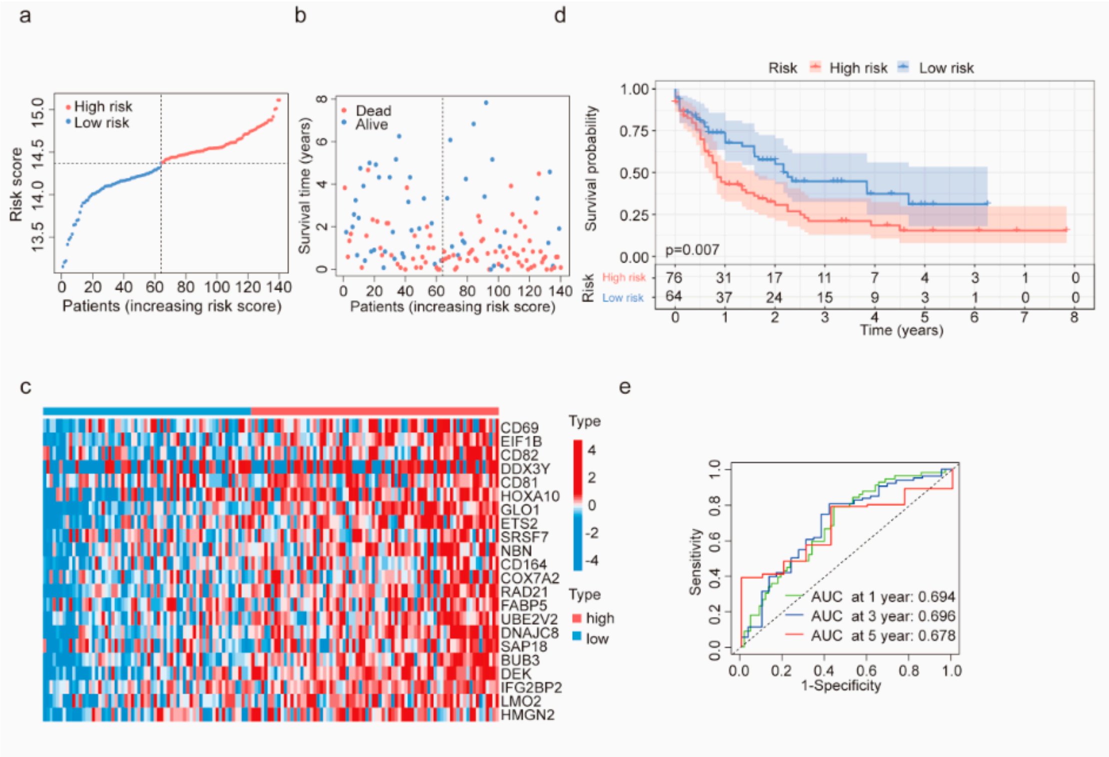

Figure S1. Use the SF-res-22 gene set to predict the prognosis of AML patients in validation cohort (VC). (**A**, **B**) The risk score distribution (**A**) and the survival outcome (SO) analysis (**B**) of the VC. (**C**) Heat map of 22 sorafenib-resistance genes significantly related to the prognosis of patients in VC. (**D**) The Kaplan-Meier survival curves of the HR and LR patients in the VC. (**E**) The time-dependent ROC analyses of the patient prognosis evaluation model to estimate the 1-, 3-, and 5-year OS of VC patients.

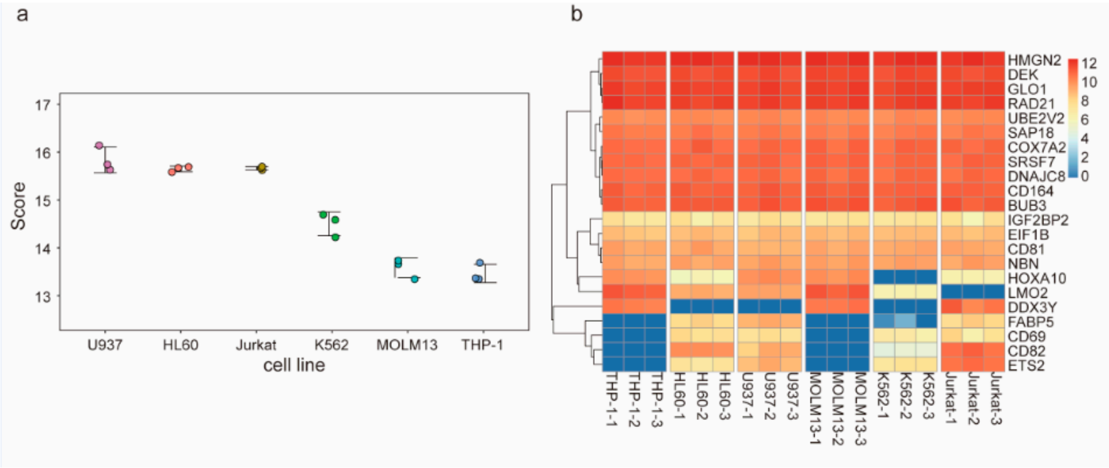

Figure S2. . Use the SF-res-22 gene set to predict sorafenib sensitivity of leukemia cells. (**A**) Analyze different leukemia cell lines in GEO database for expressions of the SF-res-22 genes to generate the sorafenib-resistance score. (**B**) Heat map of sorafenib-resistance genes in different cell lines.
