# Supplementary figures and images for: Venetoclax Overcomes Sorafenib Resistance in Acute Myeloid Leukemia by Targeting BCL2
Source: Biology (Basel). 2023 Oct 16;12(10):1337. doi: 10.3390/biology12101337 (PMC10603903; doi:10.3390/biology12101337)

Western blots in Figure 5

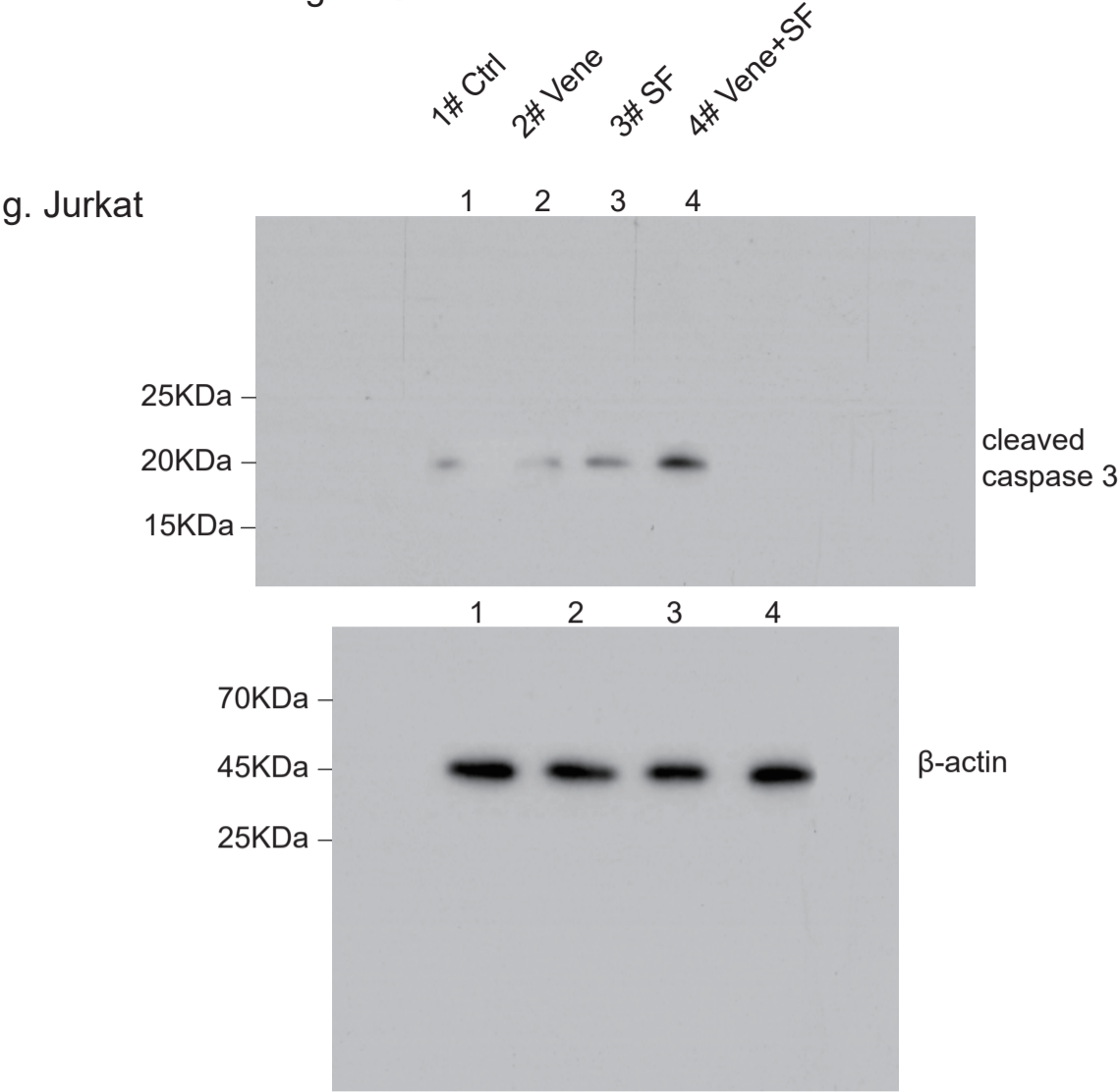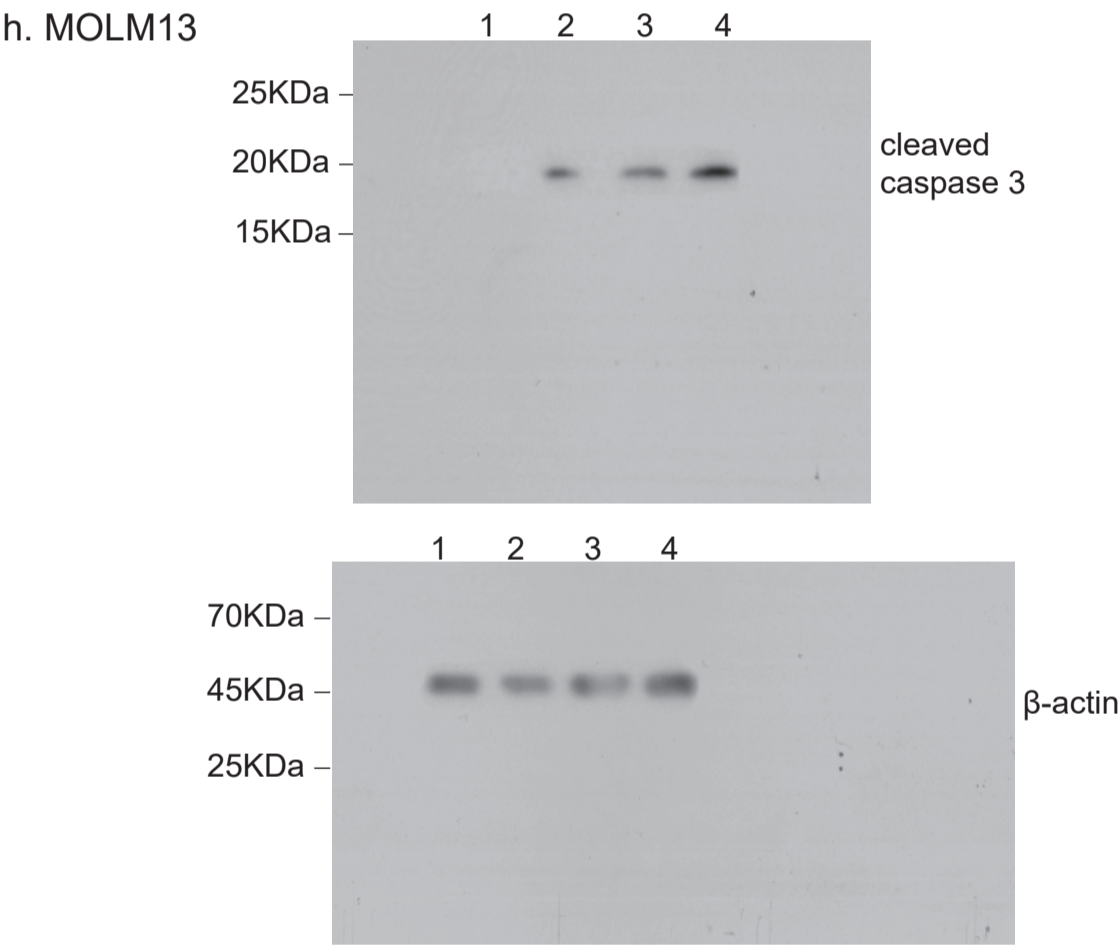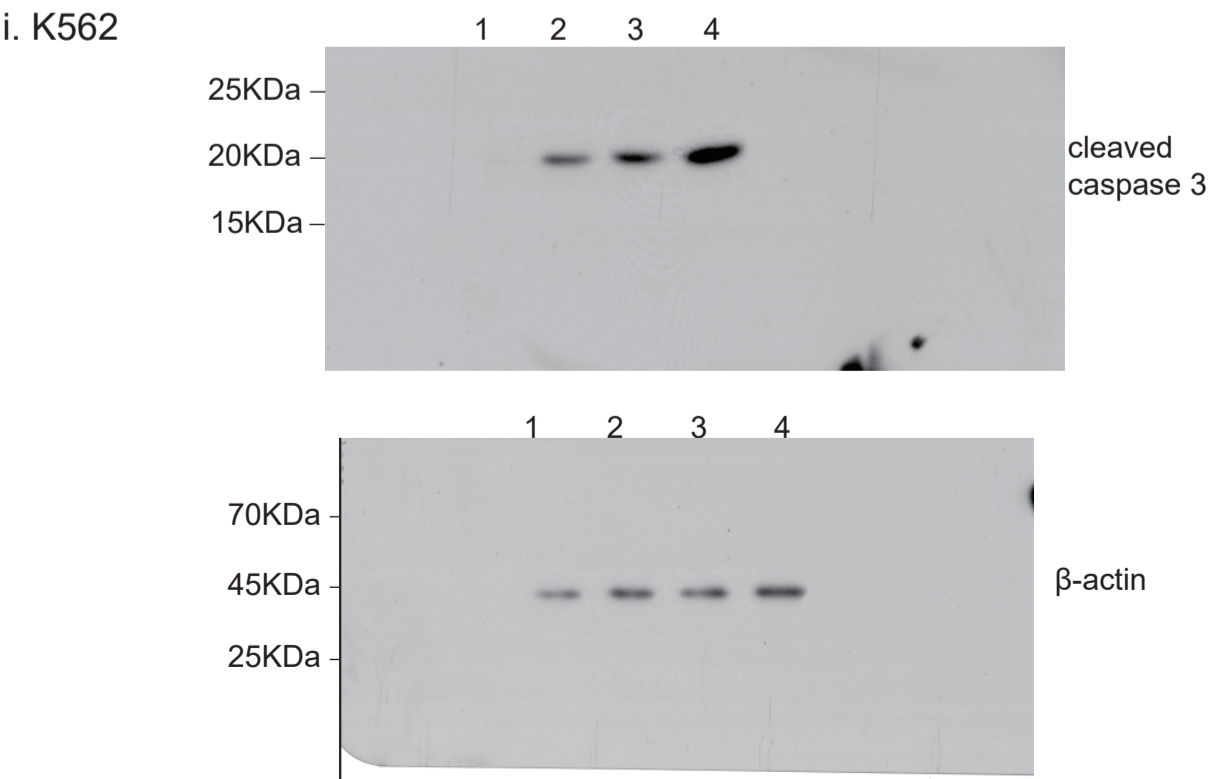

Supplement: Supplementary file 1 [file biology-12-01337-s001.zip › Figure5-WB_unjust.pdf]

Western blots in Figure 6

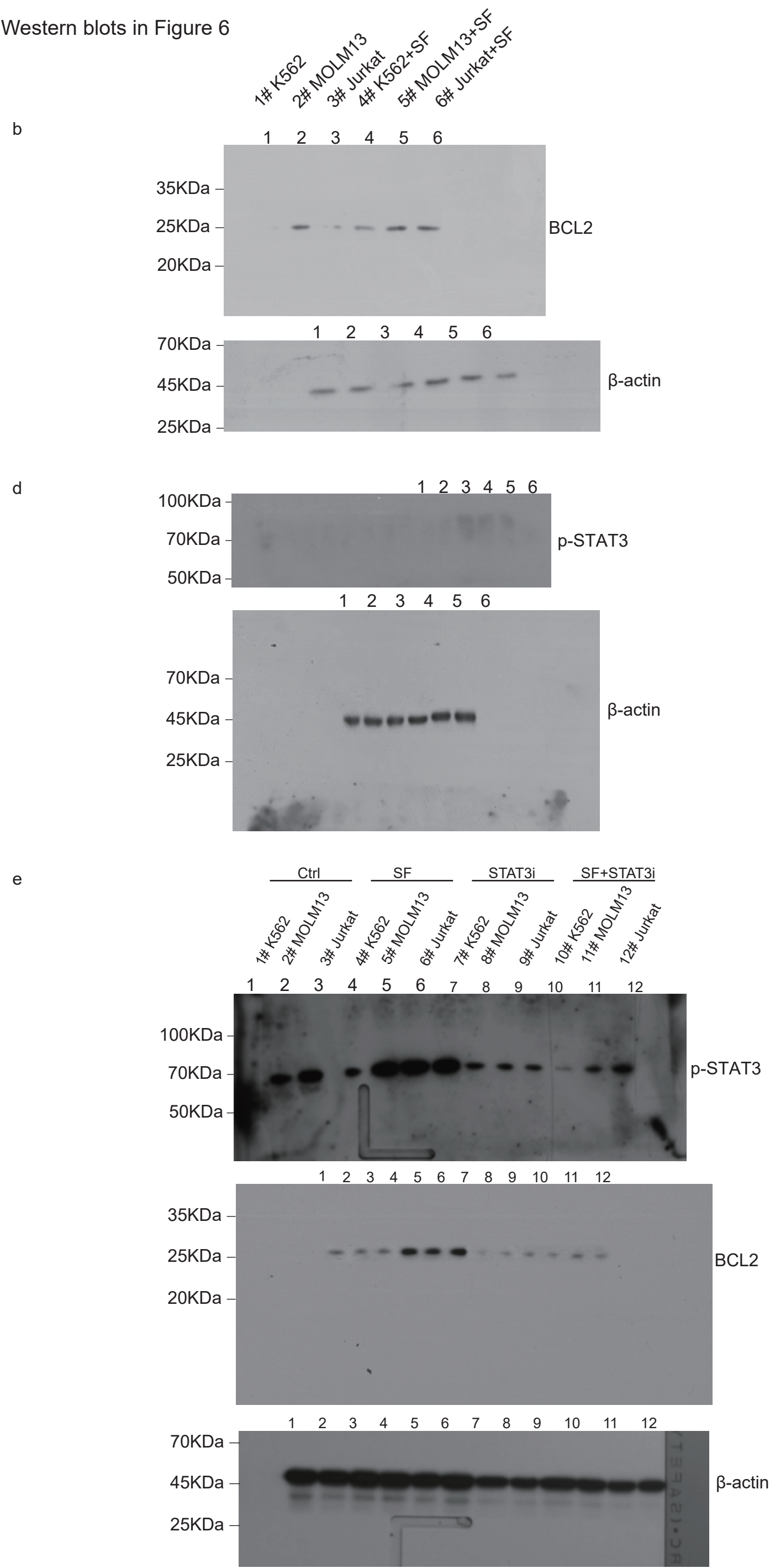

Supplement: Supplementary file 1 [file biology-12-01337-s001.zip › Figure6-WB_unjust.pdf]
